# Establishment of a sentinel surveillance network for sexually transmissible infections and blood borne viruses in Aboriginal primary care services across Australia: the ATLAS project

**DOI:** 10.1186/s12913-020-05388-y

**Published:** 2020-08-20

**Authors:** Clare Bradley, Belinda Hengel, Katy Crawford, Salenna Elliott, Basil Donovan, Donna B. Mak, Barbara Nattabi, David Johnson, Rebecca Guy, Christopher K. Fairley, Handan Wand, James Ward, and the Centre of Research Excellence in Aboriginal Sexual Health investigator group, David Lewis, Frank Bowden, Christine Selvey, Lisa Bastian, Gracelyn Smallwood

**Affiliations:** 1 South Australian Health and Medical Research Institute, North Terrace, Adelaide, SA 5000 Australia; 2 Flinders University, Sturt Rd, Bedford Park, Adelaide, SA 5042 Australia; 3 Kirby Institute, University of New South Wales, Wallace Wurth Building, High Street, Kensington, NSW 2052 Australia; 4 Kimberley Aboriginal Medical Services, 12 Napier Terrace, Broome, WA 6725 Australia; 5 Sydney Sexual Health Centre, Sydney Hospital, Macquarie St, Sydney, NSW 2000 Australia; 6 University of Notre Dame, 32 Mouat St, Fremantle, WA 6160 Australia; 7 University of Western Australia, 35 Stirling Highway, Perth, WA 6009 Australia; 8 Aboriginal Health Council of South Australia, 220 Franklin St, Adelaide, SA 5000 Australia; 9 Monash University, Wellington Rd, Clayton, VIC 3800 Australia; 10 Melbourne Sexual Health Centre, 580 Swanston St, Carlton, VIC 3053 Australia

**Keywords:** Aboriginal and Torres Strait Islander, First Peoples, Primary Care, Sexually Transmissible Infection, Blood-borne Virus, Surveillance, Reporting

## Abstract

**Background:**

Sexually transmissible infection (STI) and blood-borne virus (BBV) diagnoses data are a core component of the Australian National Notifiable Diseases Surveillance System (NNDSS). However, the NNDSS data alone is not enough to understand STI and BBV burden among priority population groups, like Aboriginal and Torres Strait Islander people, because it lacks testing, treatment and management data. Here, we describe the processes involved in establishing a STI and BBV sentinel surveillance network representative of Aboriginal Community-Controlled Health Services (ACCHS)—known as the ATLAS network—to augment the NNDSS and to help us understand the burden of disease due to STI and BBV among Aboriginal and Torres Strait Islander peoples.

**Methods:**

Researchers invited participation from ACCHS in urban, regional and remote areas clustered in five clinical hubs across four Australian jurisdictions. Participation agreements were developed for each clinical hub and individual ACCHS. Deidentified electronic medical record (EMR) data relating to STI and BBV testing, treatment and management are collected passively from each ACCHS via the GRHANITE^tm^ data extraction tool. These data are analysed centrally to inform 12 performance measures which are included in regular surveillance reports generated for each ACCHS and clinical hub.

**Results:**

The ATLAS network currently includes 29 ACCHS. Regular reports are provided to ACCHS to assess clinical practice and drive continuous quality improvement initiatives internally. Data is also aggregated at the hub, jurisdictional and national level and will be used to inform clinical guidelines and to guide future research questions. The ATLAS infrastructure can be expanded to include other health services and potentially linked to other data sources using GRHANITE.

**Conclusions:**

The ATLAS network is an established national surveillance network specific to Aboriginal and Torres Strait Islander peoples. The data collected through the ATLAS network augments the NNDSS and will contribute to improved STI and BBV clinical care, guidelines and policy program-planning.

## Background

Aboriginal and Torres Strait Islander (hereafter, respectfully, Aboriginal) people represent 3% of Australia’s total population [1] and are recognised as the First Peoples of Australia. The overall health status of Aboriginal peoples is poor in comparison to the non-Aboriginal population, an inequality largely driven by the ongoing impact of colonisation and poor progress in achieving equitable outcomes in social, cultural and economic determinants of health [2].

Aboriginal people are identified as a priority population for control of sexually transmissible infections (STI) and blood-borne viruses (BBV) because the burden of these diseases among Aboriginal peoples is much higher than in other populations in Australia [3]. Diagnosis rates of chlamydia and gonorrhoea are reported at between 3–5 and 3–30 times higher than those for non-Aboriginal Australians, respectively. An ongoing syphilis outbreak spanning four jurisdictions and amassing over 2400 cases since 2011 continues to predominantly affect Aboriginal heterosexual people aged 16–29 years living in remote communities of Australia [4]. Further, increasing notification rates of both hepatitis C and HIV among Aboriginal people are of concern, given that over the last five years, diagnoses rates among non-Aboriginal people have stabilised or decreased [3]. Accordingly, proactive approaches to diagnose and treat STI and BBV in Aboriginal populations are required.

As in many other settings, our current understanding of STI and BBV epidemics in Australia is deduced from diagnoses data alone. Once a diagnosis is made for a notifiable infection (e.g. chlamydia, gonorrhoea, trichomonas, HIV, viral hepatitis), it is a legislative requirement of medical officers or laboratories to report to jurisdictional health departments. These data are then aggregated and reported by key demographic characteristics, including Aboriginal status, periodically at the jurisdictional level and nationally. While it is acknowledged that these systems are an important component of infectious diseases surveillance in Australia, there are several constraints of the system. Most notably, the absence of STI and BBV testing data prevents the ability to contextualise changes in diagnoses data or, in the case of hepatitis C, rates of cure. Further, the system is not complete for Aboriginal status in some of the more populous jurisdictions, and thus may be inaccurately reporting the burden of disease [3, 5]. Together, these limitations impact the use of the NNDSS in assessing the burden of disease among populations, the impact of public health and clinical interventions, or for assessing clinical care and management.

Primary health care services in Australia diagnose most STI and BBV nationally and are supported by a comprehensive set of clinical guidelines at both a jurisdictional and national level [6–8]. Accordingly, primary care services routinely collect testing, diagnosis, treatment and management data related to STI and BBV. These data are an incredibly valuable but underutilised resource and can be used to help drive clinical and public health interventions [9], as well as provide greater understanding of epidemics, especially among priority populations.

A strength of the Australian healthcare system is the extensive network of Aboriginal Community-Controlled Health Services (ACCHS), established to deliver culturally appropriate health care for Aboriginal people [10]. There are over 140 ACCHS across Australia, providing a diverse range of primary care services, spanning medical care, allied health, health promotion and outreach services, to Aboriginal communities. It is estimated that ACCHS provide more than 2.5 million episodes of care annually to around 50% of the total national Aboriginal population [11]. Accordingly, participation of ACCHS is a critical component of any research activity seeking to address disease burden in Aboriginal communities.

In this paper we describe the establishment of a sentinel STI and BBV surveillance network of ACCHS and other relevant primary care services in Australia—known as the ATLAS network.

## Methods

### ATLAS network objective and rationale

The objective of the ATLAS network is to collate data to supplement the NNDSS and contribute to improved understanding of local, regional and national patterns of clinical care of STI and BBV among Aboriginal people.

### Participating research partners

**Fig. 1:**
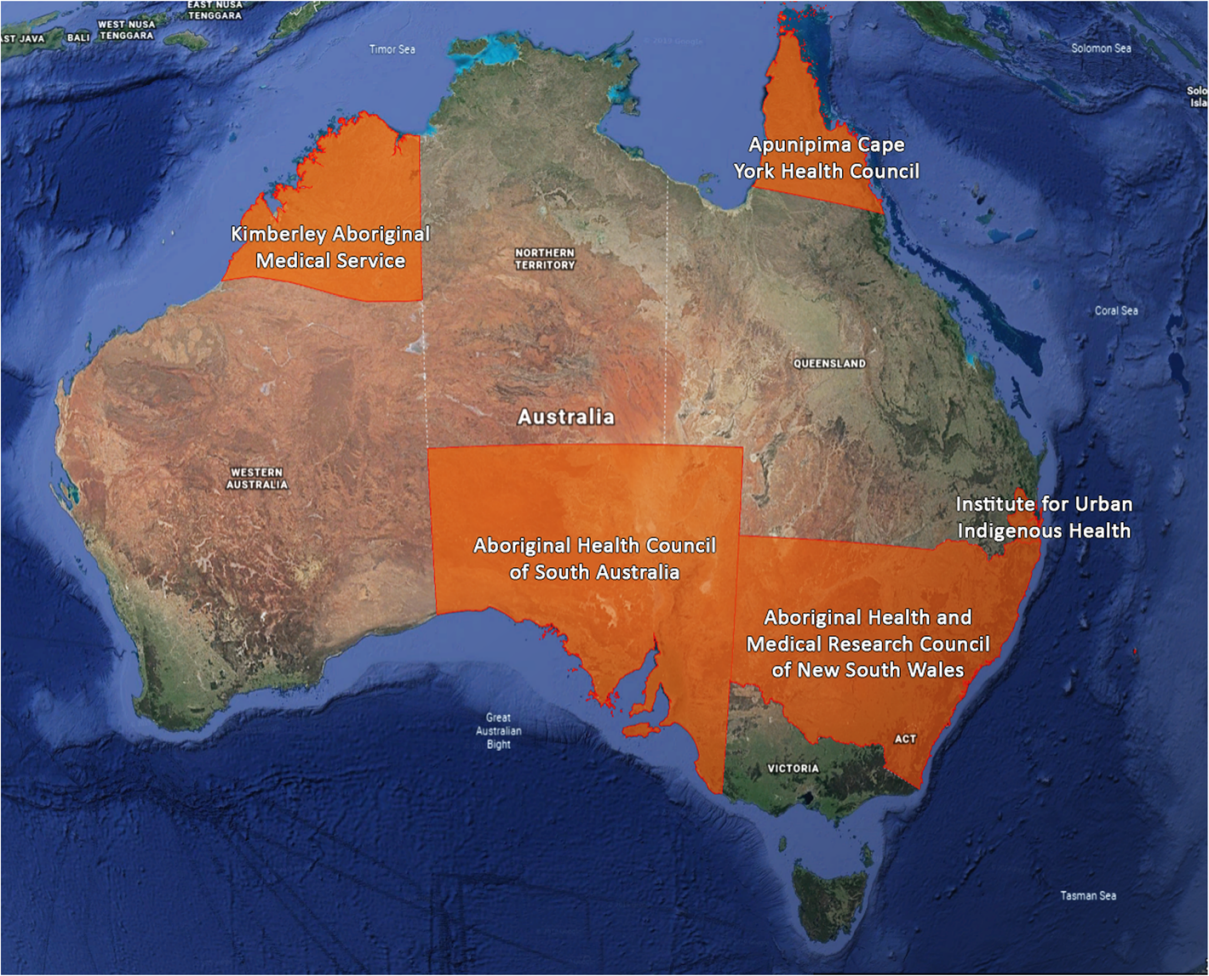
Map of CRE-ASH /ATLAS clinical hubs (image adapted from Google Earth v7.3 [12]. Map data: NASA, TerraMetrics©2020. Used in accordance with http://www.google.com/permissions/geoguidelines/)

We invited five peak Aboriginal health organisations representing regional areas in four Australian jurisdictions to partner in this research. The four jurisdictions include Queensland, South Australia, New South Wales and Western Australia. Each organisation represents the views of multiple ACCHS within their geographical remit and agreed to act as ‘clinical hubs’ for this research. The five clinical hubs are Apunipima Cape York Health Council in northern Queensland; the Institute of Urban Indigenous Health (IUIH) in south-eastern Queensland; the Aboriginal Health and Medical Research Council of New South Wales; the Aboriginal Health Council of South Australia and the Kimberley Aboriginal Medical Services (KAMS) in Western Australia (Fig. 1). These hubs were chosen based on convenience, geographic location and existing collaborative relationships with the research team. Each clinical hub is committed to oversight of the ATLAS network and other research generated as part of the study. It is our intention that the network expand over time.

### Site engagement

The research team has committed to an extensive and ongoing community and site engagement process, which commenced while the funding proposal was being developed. Executive staff, sexual health/population health specialist staff and other management from the five clinical hubs and individual ACCHS were consulted about their organisation’s participation in the ATLAS network. Each ACCHS’s governing Board approved participation in the network.

### Research governance and ethics

Formal approvals have been obtained from six Human Research Ethics Committee (HRECs) and regional research governance groups to date, including three Aboriginal-specific HRECs: the Aboriginal Health Research Ethics Committee in South Australia (EC00185, approval 04–17-732); the Aboriginal Health and Medical Research Ethics Committee (EC00342, approval 1300/17); and the Western Australian Aboriginal Health Ethics Committee (EC00292, approval 805, following approval from the Kimberley Aboriginal Health Planning Forum’s Research Subcommittee). Permissions were also obtained from the Far North Queensland Human Research Ethics Committee (as Queensland Health provides much of the primary care services in Aboriginal communities where the Apunipima Cape York Health Council is also active—EC00157, approval HREC/17/QCH/102–1173) and the Flinders University Social and Behavioural Research Ethics Committee (as the Lead Investigator’s affiliated institution—EC00194, approval OH-00142).

A Clinical Hub Reference Group, consisting of representatives from each clinical hub, provides critical oversight of and input to all research activities.

### Operation of the ATLAS network

The ATLAS network routinely collects clinical data from participating ACCHS via their electronic medical record (EMR). ACCHS in the network contribute deidentified patient records relating to clinical care (testing, treatment and management) for in-scope STI and BBV either directly from the ACCHS’s EMR or via the third-party data extraction tool GRHANITE^tm^. EMR generally do not have the capacity to extract and analyse population-level STI and BBV data internally, sufficient to evaluate clinical practice and inform Continuous Quality Improvement (CQI). The ATLAS network provides this capacity.

Most EMR require the use of third-party software to perform the extraction and delivery of relevant data in a standardised manner. We explored several different commercially-available data extraction tools—including software developed by the Improvement Foundation [13] and Pen CS [14]—before opting for the University of Melbourne’s GRHANITE software [15]. GRHANITE offered significant advantages over other software products including; compatibility with most of the EMR used in the ACCHS sector, capacity to deliver line-listed data, a substantial history of use by other sexual health research and surveillance projects [16–18], and best-practice approaches to patient deidentification and data encryption [9, 19].

For our network, the hash-based deidentification process was a key feature as it is thought to be more secure than the Australian Government’s SLK581 [20], retaining no element of personal information in the hashed identifier, yet still facilitates person-based linkage across the ATLAS network irrespective of the clinic from which the data originate. Similarly, the GRHANITE hashed identifier also can facilitate linkage to other projects and/or health services using GRHANITE. Moreover, the highly automated data collection process reduces workload for the research team once the surveillance infrastructure is established and enables further automation of the analysis and reporting system.

The initial data extraction from an ATLAS site collects data from 1 January 2016 to the date of extraction, followed by ongoing, regular (ideally, weekly) extractions. Deidentified EMR data are transferred to the ATLAS project’s secure databank located at the South Australian Health and Medical Research Institute (SAHMRI) and cleaned and stored for analysis. The ATLAS data processes utilise a custom-built SQL server accessing R Studio and Stata scripts interfacing with MS Word to produce standardised analyses for all sites participating in the surveillance network.

### Data analysis

The STI currently included in the ATLAS project are chlamydia, gonorrhoea, trichomonas, syphilis and HIV. The BBV currently included in ATLAS are hepatitis B and hepatitis C (in addition to HIV).

An initial suite of 12 STI and BBV performance measures have been developed for the ATLAS network’s surveillance reports using a co-design process with the clinical hub network (see Table 1). These performance measures are reflective of national clinical guidelines [6, 7], have a strong basis in previous research in the sector [21–28] and align with the Fifth National Aboriginal and Torres Strait Islander Blood Borne Viruses and Sexually Transmissible Infections Strategy 2018–2022’s goals regarding surveillance of existing and emerging STI and BBV issues and challenges among Aboriginal communities [29]. Refinement of these performance measures is an iterative and ongoing process conducted in collaboration with clinical hubs and services contributing to the ATLAS project but is necessarily limited to surveillance of standard clinical items that can be extracted from patient management systems.

**Table 1:** ATLAS surveillance reporting Performance Measures

Performance measures	Definition
1. **STI Testing Rate**	Proportion of clients tested for STIs (CT, NG, TV, syphilis and HIV) during the reporting period
2. **STI Testing Coverage**	Proportion of current clients tested for STIs at least once in a 12-month period
3. **STI test positivity**	Proportion of clients with at least one positive STI test in a 12-month period
4. **Completeness of STI Testing**	Proportion of clients with a positive CT and/or NG and/or TV result also tested for syphilis and HIV within 30 days of date of initial specimen collection
5. **STI Treatment Interval**	Time (days) from date of positive STI (CT, NG, TV) test to date of treatment
6. **STI Retesting Rate**	Proportion of clients retested at approximately three months (60 to 120 days) following treatment for an initial positive STI (CT/NG/TV) result
7. **STI Repeat Positivity Rate**	Proportion of clients retested at approximately three months (60 to 120 days) after treatment for an initial positive CT/NG result and who retested positive for CT/NG at this time
8. **Hepatitis B Virus (HBV) Testing Rate**	Proportion of clients receiving an HBV test. Among those testing negative, the proportion who subsequently received one or more dose of vaccination.
9. **Hepatitis C (HCV) Testing Rate**	Proportion of clients receiving an HCV antibody test and among those testing positive, the proportion then tested for RNA or viral load.
10. **HCV Treatment Uptake**	Proportion of HCV RNA positive clients prescribed Direct Acting Antiviral (DAA) treatment
11. **HCV Sustained Virological Response**	Proportion of clients who, after having been prescribed DAA treatment, achieve an undetectable viral load
12. **HPV Screening Rate**	Proportion of female clients screened for human papillomavirus in line with national guidelines

Clients: all patients aged 15 years or older attending the health service for a clinical encounter or consultation with a medical doctor, nurse or Aboriginal health practitioner

Abbreviations: *CT Chlamydia trachomatis*, *DAA* Direct Acting Antiviral, *HBV* Hepatitis B Virus, *HCV* Hepatitis C Virus, *HIV* Human Immunodeficiency Virus, *HPV* Human Papillomavirus, *NG Neisseria gonorrhoeae*, *STI* Sexually Transmissible Infection, *TV Trichomonas vaginalis*

Baseline reports addressing the current 12 performance measures, including their sub-analyses and disaggregations, were calculated and drafts provided to clinical hub/health service staff for feedback before finalisation. Suggestions for improvements were immediately incorporated into our analysis and reporting templates where feasible and we continue to refine our processes based on ongoing feedback to maximise utility for the participating sites as the surveillance network matures.

## Results

### Site engagement

To date, 29 ACCHS have joined the ATLAS surveillance network, representing 34 individual clinics and all five clinical hubs. Accordingly, participating ACCHS represent a wide geographical spread, covering metropolitan, regional, remote and very remote communities. ATLAS currently includes a slightly lower proportion of ACCHS from regional communities than would be expected from the population of all Aboriginal-specific primary care services [11]. Patient populations for these ACCHS range from 420 to 11,200 clients (Table 2).

**Table 2:** Current site engagement descriptors for ATLAS STI and BBV surveillance network

Clinical Hub	ACCHS N	Patient Populations Range
Apunipima Cape York Health Council	11	450–4470
Institute of Urban Indigenous Health	2	4820–7640
Aboriginal Health and Medical Research Council of New South Wales	3	3700–4830
Aboriginal Health Council of South Australia	6	320–7800
Kimberley Aboriginal Medical Services	7	420–11,220

Some concerns, primarily relating to the integrity of clinical data being extracted from patient records, and safety and storage of data extracted from EMR offsite for analysis, were raised by individual ACCHS and clinical hub representatives during site engagement consultations. Conversely, ACCHS and clinical hubs welcomed positive aspects of the network, including: the ability to have patient data collated at the ACCHS level; the opportunity to participate in the development of the 12 performance measures; the ability to benchmark their service with similar primary health care services; and having data available for STI and BBV to integrate within other CQI activities already underway within their health service.

### Data extraction from ACCHS

Four different EMR have been encountered in the ATLAS network to date: CommuniCare^tm^, Best Practice^tm^, Medical Director^tm^ and MMEx^tm^. The first three of these EMR do not have the capacity to readily extract deidentified line-listed patient data for specific research projects and required development of specific GRHANITE interfaces to facilitate this. The MMEx EMR, the principal system used by ATLAS’s IUIH (Brisbane) and KAMS (Kimberley) clinical hubs, is able to extract and export deidentified line-listed data in the form of .csv files via the proprietary ISA Insights reporting tool. Nevertheless, the increased privacy and linkage capabilities of the GRHANITE tool are considered to have advantages over MMEx’s internal systems and the ATLAS team has worked with GRHANITE’s developers, and key data management staff in the relevant clinical hubs, to also design and implement an interface for the MMEx EMR to take advantage of this highly desirable functionality.

### Data analysis and reporting

Irrespective of the mode of data delivery (GRHANITE or direct-export), baseline surveillance reports addressing the current 12 performance measures are developed for each ACCHS on entry to the surveillance network and ongoing surveillance reports provided at regular intervals thereafter. ACCHS that joined ATLAS early were provided with baseline reports covering 1 January 2016 to 31 December 2017 while later entrants had this baseline period brought forward to 1 January 2017 to 31 December 2018. Early baseline reports have also been updated to match this timeframe. New data are extracted at regular and ongoing intervals, and six-monthly reports are prepared for participating ACCHS approximately three months after the focal time-period occurs, to allow for record finalisation and assessment of appropriate follow-up. Newly-imported data are automatically processed to clean the data and apply transformations to align to a standard data structure. The synthesised database can then be used to produce health service-specific analyses or analyses at a more aggregated level, such as benchmarking within individual clinical hubs as well as comparisons across the ATLAS network more widely (e.g. analyses by remoteness or grouped by patient population size).

The research team are currently developing an analysis and reporting infrastructure whereby ACCHS can access a secure online dashboard and generate their own analyses and reports. This will not only give ACCHS greater control over their own data and the analyses of most relevance to their practice, it will also capitalise on the ATLAS network’s capacity to update GRHANITE extractions on a weekly basis, thus improving the timelines for return of the data.

## Discussion

The ATLAS network is one of the largest clinical surveillance networks operating in Australian ACCHS. Establishment of the ATLAS network has been a justifiably lengthy process with regular communication, consultation and feedback required of ACCHS and clinical hubs. Engagement with relevant research governance and ethical review committees across the network was also a lengthy and complex process. Five separate approval pathways needed to be followed, with the inherent complication of changes required by one research/ethics committee needing to be replicated across the network to ensure uniformity, which was often difficult to achieve when approval of the protocol was already in train or had been granted (and implemented) in other parts of the network. The achievements of the ATLAS project in the first half of its funding period must be viewed with consideration of the lengthy time required for these negotiations.

The ATLAS network returns data to health services on a regular basis, for use in evidence-based CQI processes applying a ‘plan-do-check-act’ cycle through which iterative improvements in service provision can be made [30]. The current 12 performance measures are based on national clinical guidelines and key concepts identified by previous research of the investigator group and others. These performance measures, and their sub-analyses, were further developed with the clinical hubs using a co-design approach and feedback from participating ACCHS has been positive. The ATLAS network also features a comprehensive, standardised and automated data collection and analysis infrastructure that will facilitate expansion of the network. Moreover, the use of GRHANITE and its hashed deidentifier, which cannot be reverse-engineered but is the same for the same individual in every setting, facilitates the synthesis of ATLAS data with that of other surveillance systems using GRHANITE, such as the Australian Collaboration for Coordinated Enhanced Sentinel Surveillance (ACCESS) [16] and Test Treat ANd GO2 [31] projects, will give a more complete picture of service access and health care delivery by individuals and/or by regions.

The establishment of the ATLAS surveillance network, has enabled trust to be built between the researchers and health services, and supported shared values of spirit and integrity, reciprocity, respect, equity, cultural continuity, and responsibility in all of the network’s activities [32]. Because of the sensitive nature of the research topic, trust and a highly ethical approach to data collection, management and reporting have been critical. Recognition of the ACCHS’s continued ownership of the contributed data has also been key, with an important focus of the project being to facilitate the prompt return of the data in a form readily accessible to health service staff and strengthening capacity for health services to control the analysis process.

### Strengths

The ATLAS network has several key strengths: firstly, the network of relationships built on trust and shared core values between the research team and the ACCHS sites. Without this, the surveillance system could not operate. Secondly, the large number of ACCHS participating in the project contributes critical diversity and representativeness to the surveillance system. Another key strength is the inclusion of urban and inner-regional ACCHS; the burden of STI and BBV in non-remote Aboriginal populations is largely unknown and ATLAS will be the first Aboriginal-specific surveillance network to address this knowledge gap. Expansion of our activities to include data from complementary networks will only strengthen this important component of our work.

Our development of GRHANITE and its ability to interact with MMEx is novel and substantially contributes to secure access and inclusion of this popular EMR. The parallel development of an externally-facing secure online dashboard not only addresses issues of analytic capacity in such a large and complex network but also allows for improved access to current data and customisable analyses for the ACCHS. Moreover, by automating so much of the data collection, analysis and reporting tasks, the sustainability of the ATLAS STI and BBV network is increased and the infrastructure will be easy to support beyond the life of its current funding.

### Limitations

The ATLAS STI and BBV surveillance project currently has several limitations, most of which will be overcome as the network matures. A major limitation has been the impact of the time required to establish approval for the implementation of our research. Despite long-standing relationships between the research team and the clinical hubs, significant time has had to be devoted to working with the clinical hubs to determine an acceptable approach to data collection and use and to engage ACCHS within the network. Ultimately a strength of the ATLAS project, the impact of this (ongoing) relationship-building process on project timelines must be acknowledged. Similarly, complex research governance and ethics approval processes have had a greater impact on ATLAS than would be the case for a non-Aboriginal surveillance system. National mutual acceptance of ethical review is largely inappropriate in the Aboriginal health sector, as one community cannot know or speak on behalf of the many independent and highly diverse Aboriginal communities across the country. Accordingly, in a national research project, a large number of research governance groups and ethics committees must be approached, which leads to complex cycles of feedback and amendments and substantially increases the time to establishment for networks like ATLAS.

More technical limitations have also been encountered, such as the impact of staff turnover within the SAHMRI-based team as well as some—but not all—of our partnering and participating organisations. Other issues are associated with the difficulties of designing and developing a robust data infrastructure to collect and synthesise data sourced from a large number of EMR systems, all of which seem to be used by each ACCHS in a slightly different manner. Again, the impact of these limitations will largely reduce as the ATLAS system matures and, as in the case of issues associated with the diversity of EMR in the network, will ultimately prove to be a strength of the established surveillance network.

Finally, as the ACCHS were not randomly selected, the data is not representative of all ACCHS and will not reflect testing, treatment and management data for all Aboriginal communities in Australia. We do not currently have reliable data to determine if there are significant differences between the ACCHS that have been included in this surveillance network and those that have not. However, participating ACCHS represent a wide geographical spread of ACCHS, including metropolitan, regional, remote and very remote communities. In addition, the surveillance network has been developed in such a way that it can be easily expanded in the future to include more ACCHS and improve representation.

## Conclusion

The ATLAS surveillance network is a unique data infrastructure addressing an important knowledge gap in the Aboriginal health sector. ATLAS will make an important contribution to improved understanding of local, regional and national patterns of STI and BBV to inform clinical practice, policy, and program planning and implementation. ATLAS is already strengthening relationships between ACCHS and researchers; this strengthening is bound to consolidate as the information exchange process matures.

## Data Availability

The data that support the findings of this study remain the property of the participating ACCHS and so are not publicly available. However, data may be available upon reasonable request and with explicit permission of the participating ACCHS. Please contact the corresponding author for further information.

## Abbreviations

ACCHS: Aboriginal Community-Controlled Health Service
BBV: Blood-borne virus
CT: *Chlamydia trachomatis* (chlamydia)
CQI: Continuous Quality Improvement
DAA: Direct Acting Antiviral
EMR: Electronic medical record
HBV: Hepatitis B Virus
HCV: Hepatitis C Virus
HIV: Human Immunodeficiency Virus
HPV: Human Papillomavirus
HREC: Human Research Ethics Committee
IUIH: Institute of Urban Indigenous Health
KAMS: Kimberley Aboriginal Medical Services
NG: *Neisseria gonorrhoeae* (gonorrhoea)
NNDSS: National Notifiable Diseases Surveillance System
SAHMRI: South Australian Health and Medical Research Institute
STI: Sexually Transmissible Infection
TV: *Trichomonas vaginalis* (trichomonas)
